# Molecular surveillance of carbapenemase-producing *Pseudomonas aeruginosa* at three medical centres in Cologne, Germany

**DOI:** 10.1186/s13756-019-0665-5

**Published:** 2019-12-30

**Authors:** Elena Schäfer, Monika Malecki, Carlos J. Tellez-Castillo, Niels Pfennigwerth, Lennart Marlinghaus, Paul G. Higgins, Frauke Mattner, Andreas F. Wendel

**Affiliations:** 1 Institute of Hygiene, Cologne Merheim Medical Centre, University Hospital of Witten/Herdecke, Ostmerheimer Strasse 200, 51109 Cologne, Germany; 2 Department of Clinical Microbiology, MVZ synlab Leverkusen GmbH, Site Köln-Merheim, Cologne, Germany; 3 Department of Medical Microbiology, National Reference Centre for Multidrug-resistant Gram-negative Bacteria, Ruhr-University Bochum, Bochum, Germany; 4 Institute for Medical Microbiology, Immunology and Hygiene, University of Cologne, and German Centre for Infection Research, Partner site Bonn-Cologne, Cologne, Germany

**Keywords:** *Pseudomonas aeruginosa*, Carbapenemase, Surveillance, VIM-2

## Abstract

**Background:**

*Pseudomonas aeruginosa* is a common pathogen causing hospital-acquired infections. Carbapenem resistance in *P. aeruginosa* is either mediated via a combination of efflux pumps, AmpC overexpression, and porin loss, or through an acquired carbapenemase. Carbapenemase-producing *P. aeruginosa* (CPPA) strains are known to cause outbreaks and harbour a reservoir of mobile antibiotic resistance genes, however, few molecular surveillance data is available. The aim of this study was to analyse the prevalence and epidemiology of CPPA in three German medical centres from 2015 to 2017.

**Methods:**

Identification and susceptibility testing were performed with VITEK 2 system. *P. aeruginosa* non-susceptible to piperacillin, ceftazidime, cefepime, imipenem, meropenem and ciprofloxacin (4MRGN according to the German classification guideline) isolated from 2015 to 2017 were analysed. A two-step algorithm to detect carbapenemases was performed: phenotypic tests (EDTA- and cloxacillin-combined disk tests) followed by PCR, Sanger sequencing, and eventually whole genome sequencing. CPPA isolates were further genotyped by RAPD and PFGE. In-hospital transmission was investigated using conventional epidemiology.

**Results:**

Sixty two *P. aeruginosa* isolates were available for further analysis, of which 21 were CPPA as follows: *bla*_VIM-1_ (*n* = 2), *bla*_VIM-2_ (*n* = 17), *bla*_NDM-1_/*bla*_GES-5_ (n = 1) and the newly described *bla*_IMP-82_ (n = 1). CPPA were mostly hospital-acquired (71.4%) and isolated on intensive care units (66.7%). All (except one) were from the tertiary care centre. PFGE typing revealed one large cluster of VIM-2-producing CPPA containing 13 isolates. However, using conventional epidemiology, we were only able to confirm three patient-to-patient transmissions, and one room-to-patient transmission, on several intensive care units.

**Conclusions:**

These data give insight into the epidemiology of CPPA in three centres in Germany over a period of 3 years. Carbapenemases are a relevant resistance mechanism in 4MRGN-*P. aeruginosa,* illustrated by genetically related VIM-2-producing strains that seem to be endemic in this region. Our data suggest that infection control measures should especially focus on controlling transmission on the ICU and support the need for a local molecular surveillance system.

## Background

*Pseudomonas aeruginosa* is a leading nosocomial pathogen and infections can be difficult to treat because of rapid resistance development. The emergence of multidrug-resistant (MDR) isolates is a serious public health threat and often affects immunocompromised patients within special units (intensive care units (ICU), haematology-oncology wards or burn units) [1–4]. Resistance to carbapenems is mediated either by intrinsic resistant mechanisms (a combination of efflux pumps, AmpC overexpression and porin loss) or acquisition of a carbapenemase, especially a metallo-β-lactamase (MBL) [5]. Carbapenemase-producing *P. aeruginosa* (CPPA) isolates harbour antimicrobial resistance genes located on mobile genetic elements (mainly integrons, transposons or plasmids) that can spread to other bacteria [6–8], so microbiological monitoring and infection control surveillance is of utmost importance. Prevalence of CPPA among MDR *P. aeruginosa* differs greatly between regions, with VIM- and IMP-family carbapenemases being the most widespread [9, 10]. Additionally, CPPA are known to cause protracted outbreaks, e.g. IMP-8 or GIM-1-producing types [11, 12]. However, there is little surveillance data available combining molecular and epidemiological information. The aim of this study was to analyse the prevalence and epidemiology of CPPA in three German medical centres isolated from 2015 to 2017.

## Methods

### Setting and screening strategy

The Institute of Hygiene at the Cologne Merheim Medical Centre provides an infection control service for three medical centres in Cologne (one tertiary care centre, 700 beds; one secondary care centre, 400 beds; one children hospital, 260 beds) with a total of seven ICUs between them. Microbiological specimens are sent to the private microbiology laboratory MVZ synlab Leverkusen. The protocol of the German healthcare-associated infection surveillance on intensive care units (ITS-KISS) was followed on all seven ICUs during the study period [13]. The number of patients colonized/infected with MDR *P. aeruginosa* was assessed using the laboratory surveillance information system (Hybase v.6, epiNET AG, Germany). A risk-based rectal admission screening on multidrug-resistant Gram-negative organisms was performed in the three hospitals (stay at a healthcare facility abroad or on a German ICU within the last year, known positive carrier status or contact to other patients carrying carbapenem-resistant Gram-negative organisms). On most intensive care units (five out of seven) a general admission screening was implemented.

### Identification and susceptibility testing

All inpatient isolates were identified with standard microbiological procedures using the VITEK 2 system (Vitek GN-ID, bioMérieux, Marcy l’Etoile, France) or MALDI-TOF (Bruker Daltonics, Bremen, Germany). Susceptibility testing was performed with the VITEK 2 system (Vitek AST-N248). EUCAST breakpoints were used for interpretation (v.8.0, May 2018). *P. aeruginosa* non-susceptible (intermediate or resistant) to piperacillin, ceftazidime, cefepime, imipenem, meropenem and ciprofloxacin (4MRGN according to the German classification guideline for Gram-negative multidrug-resistant organisms [14], at least MDR according to ECDC/CDC classification [15]) isolated from clinical and screening specimens from 2015 to 2017 were included. Bacterial isolates were stored in a 30%-glycerol stock at − 20 °C.

### Phenotypic and molecular screening and detection of carbapenemases

A two-step algorithm to detect carbapenemases was performed, comprised of phenotypic and genotypic tests. We performed two combined disk tests (CDT) using (a) 10 μg imipenem with or without 930 μg EDTA and (b) 10 μg imipenem with or without 4000 μg cloxacillin. A difference of (a) ≥ 5 mm or (b) < 6 mm in zone diameter was considered to be indicative of (a) an MBL [16] or (b) a carbapenemase [17]. Quality controls with strains provided by the German National Reference Centre for Multidrug-resistant Gram-negative Bacteria were performed. CDT-positive isolates were further confirmed by several PCRs and sequencing, first a *bla*_IMP_/*bla*_VIM_ duplex PCR [16, 18], followed by screening for the *bla*_GIM-1_, *bla*_NDM_, *bla*_KPC_, *bla*_OXA-48_ and *bla*_GES_ genes [6, 19].

One IMP-producing isolate was further examined by whole genome sequencing because we were unable to assess the exact *bla*_IMP_-type by conventional sequencing. Total DNA was isolated using the MagAttract HMW DNA Kit (Qiagen, Hilden, Germany). Sequencing libraries were prepared using the Nextera XT library prep kit (Illumina GmbH, Munich, Germany) for a 250 bp paired-end sequencing run on an Illumina MiSeq sequencer. De novo assembly was performed using Velvet (version 1.1.04) [20]. An N50 of 52,548 bp was achieved. Acquired resistance genes on assembled sequences were identified by ResFinder (version 3.1; threshold of 98% identity and minimum length of 60%) [21]. Sequence reads of the newly described *bla*_IMP-82_-variant have been deposited under the nucleotide accession number GenBank MN057782.

### Genotyping

Carbapenemase-positive isolates were first genotyped by RAPD (three primers: ERIC-1, ERIC-2 and ST272 [22]). Isolates differing by one or more bands were assigned to distinct types. Genotyping was additionally carried out by PFGE after *Bcul*I/*Spe*I (New England BioLabs, USA) restriction under the following conditions: 6 V/cm for 24 h with pulse times of 5 s to 33 s at 14 °C. The strain relatedness was calculated with the BioNumerics Tree and Network Inference Module (version 7.6) using band-based Dice similarity coefficient and the unweighted pairs geometric-matched analysis dendogram (band matching tolerance 0.5% and optimization 0.5%) in accordance with the Tenover et al. criteria [23]. The cut-off value to define a PFGE cluster was set at ≤6 band differences (corresponding to equal or less than two genetic events) and 76%.

### Infection prevention and control analysis

Relevant clinical and epidemiological data were collected by an infection control nurse. Bacterial isolates and infections were considered as community-acquired if the collection of the specimen or the start of infection occurred on or before the 2nd day of admission. Thereafter, bacterial isolates and infections were defined as hospital-acquired. Transmission analysis was based on epidemiological data (direct room or ward contact, and/or documented care by the same staff) and genetic data. Proven transmission events were defined as isolation of genetically-related isolates from two patients who were on the same ward at the same time (at least 24 h, patient-to-patient transmission) or in the same room with a maximum time interval of 6 months (room-to-patient transmission). An interval of 6 months was chosen because transmission of *P. aeruginosa* from environmental sources can last over longer periods and can be sporadic [11]. Hospital-acquired infections were classified according to the CDC definitions [24].

## Results

### Isolate and patient characteristics

**Fig. 1:**
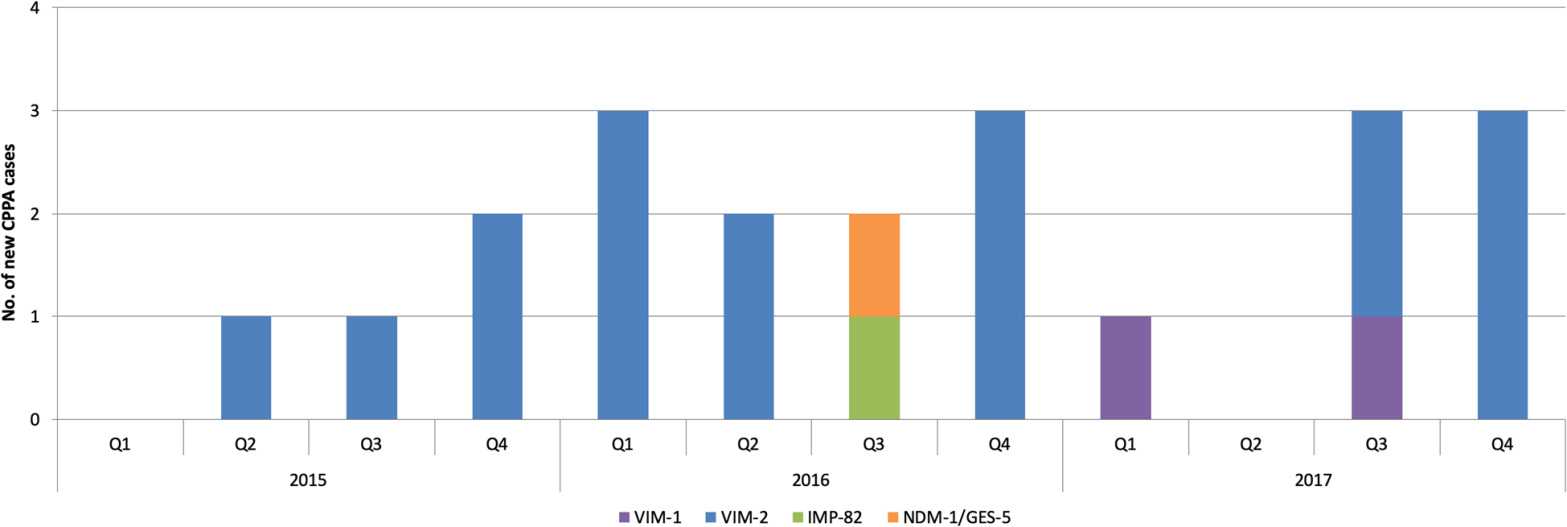
Overview of new cases with CPPA from 2015 to 2017 (Q = quarter year)

Sixty two out of 96 non-duplicate MDR *P. aeruginosa* patient isolates were available for further analysis. Molecular analysis confirmed 21 MBL-test- and cloxacillin-test-positive isolates as CPPA as follows: *bla*_VIM-1_ (*n* = 2), *bla*_VIM-2_ (*n* = 17), *bla*_IMP-82_ (*n* = 1) and *bla*_NDM-1_/*bla*_GES-5_ (*n* = 1) (Fig. 1). Four cloxacillin-test-positive and MBL-test-negative isolates were not confirmed as carbapenemase-producers.

All CPPA showed an extensively drug-resistant (XDR) phenotype (based on the ECDC/CDC scheme; fosfomycin was not included as there are no clinical breakpoints available according to EUCAST [15]). Fifteen out of 21 CPPA were hospital-acquired, 12 of which were from intensive care units and all except one from the tertiary care centre. Six CPPA were community-acquired. However, five out of these six affected patients received health care within the 30 days before diagnosis. Three patients were transferred to our hospital after hospital stays in Serbia (*bla*_NDM-1_), Sri Lanka and Cyprus (*bla*_IMP-82_) or Turkey (*bla*_VIM-1_).

More than half of the patients (*n* = 11) were treated in surgical departments (for trauma, burn, colon disease etc.), eight other patients in internal medicine (for heart or pulmonary disease). Most affected patients (*n* = 15) received an antipseudomonal antibiotic therapy (eight patients had more than one antipseudomonal antibiotic agent) within the 7 days before colonization/infection with CPPA as follows: carbapenems (*n* = 9), ciprofloxacin (*n* = 8), piperacillin-tazobactam (*n* = 6), ceftazidime or cefepime (*n* = 3), and colistin (*n* = 3). Relevant clinical and epidemiological data of the 21 patients colonized/infected with carbapenemase-producing *P. aeruginosa* are summarized in Table 1.

**Table 1:** Characteristics of 21 patients with carbapenemase-producing *P. aeruginosa*

Patient characteristics (*n* = 21)	Value
Age (years)	
mean	62
range	20; 80
Sex	
male	16 (76.2%)
Source of first positive specimen	
respiratory tract	7 (33.3%)
urine	5 (23.8%)
screening (rectum)	5 (23.8%)
wound	2 (9.5%)
other	2 (9.5%)
Infection/colonization with CPPA	
hospital-acquired	15 (71.4%)
community-acquired	6 (28.6%)
Day of acquisition during hospital stay (hospital-acquired CPPA only; *n* = 15)	
mean	19
range	8; 82
Medical centres	
tertiary care	20 (95.2%)
secondary care	1 (4.8%)
children hospital	0 (0%)
Ward type	
ICU	14 (66.7%)
general ward	7 (33.3%)
Medical departments	
surgery	11 (52.4%)
internal medicine	8 (38.1%)
others	2 (9.5%)
Hospital-acquired infection (CDC)	
pneumonia	5 (23.8%)
urinary tract	2 (9.5%)
skin infection	2 (9.5%)
Antipseudomonal antibiotic therapy^a^	15 (71.4%)
Surgery^a^	15 (71.4%)
Nonsurgical intervention^a^	19 (90.5%)
Dialysis^a^	6 (28.6%)
Mechanical ventilation^a^	16 (76.2%)
Wounds^a^	15 (71.4%)
Central line^a^	17 (80.1%)
Urinary catheter^a^	18 (85.7%)

^a^within a maximal interval of 7 days before first isolation of CPPA

### Genotyping and transmission analysis

RAPD revealed two clusters of VIM-2-producing *P. aeruginosa* containing 13 and 2 isolates each (cluster 1 and cluster 2 respectively). PFGE was only able to confirm cluster 1 (PFGE type A); the PFGE patterns of the cluster 2 isolates displayed eight band differences. All other isolates were unrelated to each other.

**Fig. 2:**
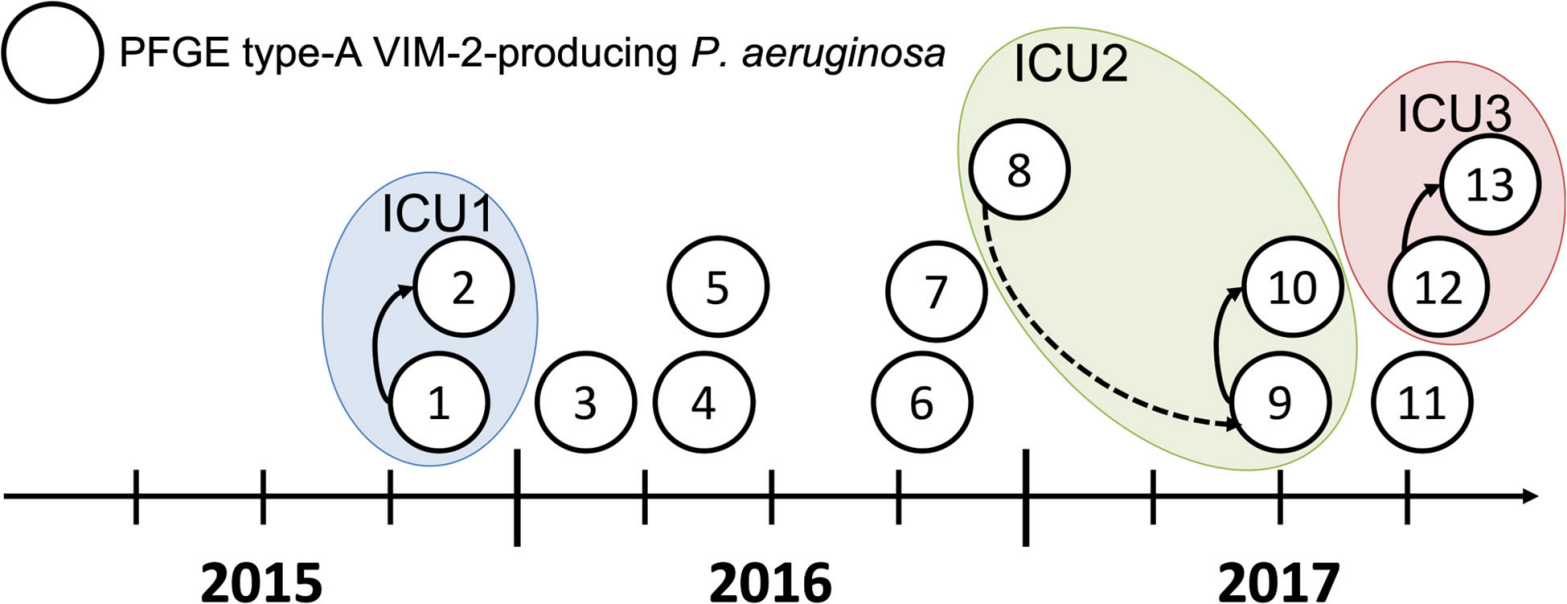
Epidemiological timeline and transmission route of PFGE type A VIM-2-producing *P. aeruginosa*. Each node represents one patient at time of first isolation. Arrow indicates genetically and epidemiological confirmed transmission events (dashed line = room-to-patient; continuous line = patient-to-patient). Encircled nodes indicate ward of transmission. Positions of the nodes on the y-axis were randomly chosen

Eleven out of 13 PFGE type A isolates were hospital-acquired. However, analysing spatiotemporal links of these patients, we were only able to confirm three patient-to-patient transmissions on three different ICUs (one in 2015 and two in 2017) and one room-to-patient transmission on an ICU in 2017. All transmissions occurred in the tertiary care centre and we were not able to define an index patient as all linked isolates were hospital-acquired (Fig. 2).

## Discussion

In contrast to carbapenem-resistant *Acinetobacter baumannii* complex or carbapenem-resistant Enterobacterales*,* carbapenemases are detected less frequently in carbapenem-resistant *P. aeruginosa* in which carbapenem-non-susceptibility is predominantly mediated by other mechanisms (a combination of efflux pumps, AmpC overexpression and porin loss) [5, 25]. However, early detection of these mobile broad-spectrum β-lactamases is necessary to prevent the propagation mainly of metallo- β-lactamases, across other Gram-negative organisms in the healthcare-setting [25, 26].

In our study, carbapenemases, mainly VIM-2, were detected in one third of the MDR/XDR *P. aeruginosa* isolates. The rate of CPPA and proportions of the different carbapenemase gene families in this study are in line with other observations. In 2017 approximately 27.7% of the *P. aeruginosa* isolates referred to the German reference centre carried a carbapenemase, VIM-2 being by far the most prevalent one [27]. In a German multicentre study, 32% of the carbapenem-resistant *P. aeruginosa* isolates were carbapenemase producers, with VIM-2 being the most prevalent enzyme [28]. Studies combining molecular surveillance and prevalence data at two German tertiary care centres detected a CPPA proportion of 40% in MDR isolates (all *bla*_VIM_) and 23% in XDR isolates (mostly *bla*_VIM-1_ and *bla*_VIM-2_) [29, 30]. Nevertheless, the local epidemiology can differ greatly between medical centres, e.g. in a tertiary care centre 40 km from Cologne the most prevalent carbapenemase gene in *P. aeruginosa* was *bla*_GIM-1_ [6]. In another hospital in southern Germany *bla*_IMP_ was widespread [12]. Overall, it is difficult to compare prevalence studies as bacterial isolate selection, inclusion and screening criteria, as well as test algorithms differ greatly. Until now there are no official recommendations by EUCAST addressing carbapenemase screening cut-off values in *P. aeruginosa* comparable to those existing for Enterobacterales [31]. Official screening recommendations are based on the three antibiotics imipenem, meropenem and ceftazidime (German National Reference Centre) or on imipenem, meropenem and piperacillin-tazobactam (British standards) [32, 33]. Overall, we chose a well-defined significant subgroup of MDR *P. aeruginosa* since all isolates non-susceptible to piperacillin, ceftazidime, cefepime, imipenem, meropenem and ciprofloxacin (4MRGN) directly result in infection prevention and control (IPC) measures [14].

Molecular surveillance of bacterial isolates combined with epidemiological and infection data can lead to direct implementation of targeted IPC measures. Surveillance of *P. aeruginosa* is of utmost importance as it can reside in the inanimate patient environment and subsequently lead to transmission and to colonization or infection. *P. aeruginosa* can reside in the sink drains in the patient room for long periods. The spreading and distribution of MDR *P. aeruginosa* in the shower and sink drains, and sewage system of the ward is quite complex as several studies have shown [11, 34]. We found direct and indirect evidence for both modes of transmission (patient-to-patient and room-to-patient). Although, most *bla*_VIM-2_-carrying *P. aeruginosa* isolates clustered in the PFGE analysis, we were only able to confirm a few transmission events. Interestingly, transmission happened exclusively on the intensive care units of the tertiary care centre. Therefore IPC measures should focus on the ICU, where the relevant patients at risk for colonization/infection with CPPA are found (e. g. antimicrobial therapy, prolonged hospitalization, medical devices, and severe underlying disease) [2, 12, 35]. Moreover, two out of the 13 patients who carried a related (cluster 1) CPPA at admission were referred from another hospital in the region. Thus, genetically related strains may be endemic in the region.

There are a few limitations in this study. We were not able to provide full prevalence data, as only two third non-duplicate 4MRGN isolates detected during this period were available. However, our prevalence data is in line with other studies. Secondly, we were able to detect a dominant *bla*_VIM-2_-carrying strain using PFGE; for further discrimination whole genome sequencing is needed and further studies will address this. Thirdly, our inclusion criteria were probably not sensitive enough to detect all CPPA. On the other hand, CPPA is often associated with MDR- or XDR-phenotypes, corresponding to our inclusion criteria [36]. Extending the screening inclusion criteria would lead to more negative results and clinical microbiology laboratories may not have the resources.

## Conclusions

The surveillance of MDR *P. aeruginosa* based on carbapenemase detection, genotyping and classic epidemiology revealed a relevant prevalence of VIM-2 with endemic spread of a genetically highly-related strains, and proven transmission on intensive care units. This underlines the importance of such methodology for surveillance and the results support the need for a local molecular surveillance system.

## Data Availability

Sequence reads have been deposited at the nucleotide accession number GenBank MN057782. All other data generated or analysed during this study are included in this published article.

## Abbreviations

4MRGN: Multiresistente gramnegative Stäbchen mit Resistenz gegen 4 der 4 Antibiotikagruppen (Gram-negative multidrug-resistant organisms with resistance to 4 antibiotic classes, according to the German classification guideline, see methods)
CDC: Centers for Disease Control and Prevention
CDT: Combined disk test
cgMLST: core genome multilocus sequence type
CPPA: Carbapenemase-producing *Pseudomonas aeruginosa*
ECDC: European Centre for Disease Prevention and Control
EDTA: Ethylenediaminetetraacetic acid
EUCAST: European Committee on Antimicrobial Susceptibility Testing
ICU: Intensive care unit
IPC: Infection prevention and control
ITS-KISS: Intensivstation-Krankenhaus-Infektions-Surveillance-System = German national nosocomial infections surveillance on intensive care units
MALDI-TOF: Matrix-assisted laser desorption/ionization - time-of-flight mass spectrometer
MBL: Metallo-β-lactamase
MDR: Multidrug-resistant
PFGE: Pulsed-field gel electrophoresis
RAPD: Random amplification of polymorphic DNA
XDR: Extensively drug-resistant
